# An Ethnobotanical Survey of a Dryland Botanical Garden and Its Environs in Kenya: The Mutomo Hill Plant Sanctuary

**DOI:** 10.1155/2020/1543831

**Published:** 2020-03-17

**Authors:** Fredrick Munyao Mutie, Lun-Lun Gao, Vivian Kathambi, Peninah Cheptoo Rono, Paul Mutuku Musili, Grace Ngugi, Guang-Wan Hu, Qing-Feng Wang

**Affiliations:** ^1^CAS Key Laboratory of Plant Germplasm Enhancement and Speciality Agriculture, Wuhan Botanical Garden, Chinese Academy of Sciences, Wuhan 430074, China; ^2^Sino-Africa Joint Research Center, Chinese Academy of Sciences, Wuhan 430074, China; ^3^University of Chinese Academy of Sciences, Beijing 100049, China; ^4^East Africa Herbarium, National Museums of Kenya, P.O. Box 451660-0100, Nairobi, Kenya

## Abstract

Mutomo hill plant sanctuary is a ten-hectare piece of land in Kenya listed as a botanical garden under the Botanical Gardens Conservation International, originally established in 1964 with the aim of conserving indigenous flora from destructive anthropogenic activities. This paper presents ethnobotanical documentation of medicinal plants of Mutomo hill plant sanctuary and its environs. An ethnobotanical survey was carried out in Mutomo hill plant sanctuary and its environs with 48 herbalists aged between 32 and 96 years from July 2018 to February 2019 using a semistructured open-ended questionnaire. The plants were collected through random surveys with each herbalist in different ecotypes around the villages and within the Mutomo hill plant sanctuary. The Relative Frequency of Citation (RFC) for each species reported was calculated to determine the plant species frequently collected. In total, 68 different plant species distributed in 28 families and 54 genera were reported. The frequently used plant families were Leguminosae (13 species), Lamiaceae (6 species), and Euphorbiaceae (6 species). Shrubs (37%) and trees (34%) were the dominant growth habits reported. The most cited plant species were *Cassia abbreviata* Oliv. (RFC = 0.63), *Acacia nilotica* (L.). Delile (RFC = 0.54), *Strychnos heningsii* Gilg (RFC = 0.46), and *Aloe secundiflora* Engl. (RFC = 0.31). Root (19 species) and bark (19 species) were the frequently collected plant parts. Infectious diseases (33) and digestive system disorders (24) were reported to be managed with the majority of the plant species. This study contributes to safeguarding the traditional knowledge on medicinal plants in the study area, which is useful in appreciating and acknowledging the cultural heritage of the Kamba people from the local perspective of Mutomo area in Kenya. It also adds to the knowledge base and documentation of medicinal plants, which is useful information as potential data for drug development.

## 1. Introduction

The use of plants for medicine has been practiced for many years [1]. This culture has developed through trial and error and has for a long time been passed orally from one generation to another [1, 2]. African traditional medicine is the oldest, perhaps the most diverse mode of treatment and among the less known systems of medicine in the world [2–5]. Despite the production of synthetic drugs, the utilization of natural organic healing materials for the treatment of diseases has persisted throughout the world [5]. In addition, modern healthcare techniques have been less effective in the treatment of some infectious diseases such as malaria and HIV/AIDS which have affected Africa more than any other part of the world [6]. Furthermore, the use of traditional medicine has been attributed to the high cost and inaccessibility of modern healthcare care services compared to the cheap and readily available herbal medicine [6, 7]. The traditional communities have recently begun to appreciate the effectiveness of modern healthcare services against diseases such as HIV/AIDS leading to a reduction in the number of people relying on traditional medicine [8]. A recent study reported that the use of traditional medicine for health care has declined in Africa and Asia with most of the people turning to modern health care services. For example, just 1.7% and 1.5% of the populations studied in South Africa and Ghana respectively reported consulting traditional medical practitioners in the past three years [9]. An overall decline in the use of traditional practices has also been reported elsewhere in South Africa where an analysis of nationally representative population-based surveys from 1995 to 2007 showed that the use of traditional medicine had declined to 0.1% or less in the past month from 3.6–12.7% in just over a decade [10]. It has been estimated that at least 90% of the population in Kenya has used medicinal plants for health care at some point in life [11]. However, Awiti [12] reported that only 7.56% of the respondents studied consulted “nonmodern” health care providers while just 0.15% consulted traditional healers, further suggesting that the use of traditional medicine is also on the decline in Kenya. The reduction in the use of traditional medicine is probably due to the increase and cultural acceptability of modern health care services [10]. Ethnomedicinal studies in East Africa have however revealed that medicinal plants are used by many populations for various health problems such as reproductive and gynecological problems [13, 14], management of infectious diseases such as HIV/AIDS and malaria [15–17] and as antidotes against snake bites [18, 19]. In addition, medicinal plants are sold in some urban areas as a source of income [20, 21].

In Kenya, about 80% of the landmass is covered by arid and semiarid areas [22]. Drylands of Kenya play a significant role in the country's formal and informal economy, yet they have not been incorporated in the country's conservation regimes making it difficult to get a true picture of their status and trends of their biodiversity. In addition, little support and advocacy have been directed to such areas [23]. Based on the Flora of Tropical East Africa, the flora of Kenya is comprised of 6,293 indigenous vascular plant species [24] out of which over 5,000 plant species reportedly occur in drylands [25]. An estimated 1,200 plant species in Kenya are reported to have a medicinal value [26]. In Kenya, ethnobotanical studies involving various ethnic groups have been done [16, 17, 21, 27–44]. The local communities who use natural resources have interacted with the biodiversity over the years and hence have accumulated important traditional knowledge regarding their use [40]. The aging of the older generations and the change of lifestyles that have seen the younger generations take up formal education have left much of the indigenous knowledge undocumented and on the verge of being lost [45]. In addition, ethnobotanical surveys have not been done extensively in many regions of Kenya [36]. There is therefore a need for appropriate measures to mitigate the current abuse and foster proliferation of the biodiversity in drylands for the benefit of future generations [46].

Like in many other African countries, medicinal plants in Kenya are commonly collected from the wild with poor harvesting methods such as debarking [47]. Such plants are diminishing as a result of unsustainable harvesting and destruction of habitats [48]. It is on account of such destructive human activities that the early botanists in Kenya considered the need to set aside areas for conservation of important indigenous plant species. Among such areas is the Mutomo hill plant sanctuary which was established in 1964 [49] and gazetted as a botanical garden in 1993 [50]. Despite this, plant diversity loss in such areas is still in progress and is expected to increase with the increase in the human population [46]. The Kenyan wealth of dryland biodiversity and its indigenous knowledge is not well documented [23]. There are various botanical surveys done in Kitui county such as the biodiversity of Kitui hills [46], some ethnobotanical surveys [34, 40] and a checklist of the vascular plants of Mutomo hill plant sanctuary [49]. Mutomo hill plant sanctuary is a well-known botanical garden under the Botanical Gardens Conservation International [50, 51]. Despite this, ethnobotanical surveys in Mutomo hill plant sanctuary and its environs have not been done. Since the role of botanical gardens in the conservation of useful plants including medicinal plants is well-known [52], the lack of ethnobotanical data of the sanctuary and its environs justified the need for this investigation. This study brings more understanding of medicinal plant usage and knowledge thereof to the nation as cultural and scientific heritage at a local level. It also informs about plants of economic importance to the world because it adds knowledge of these medicinal plants as potential resources for drug development.

## 2. Subjects and Methods

### 2.1. Study Area

**Figure 1:**
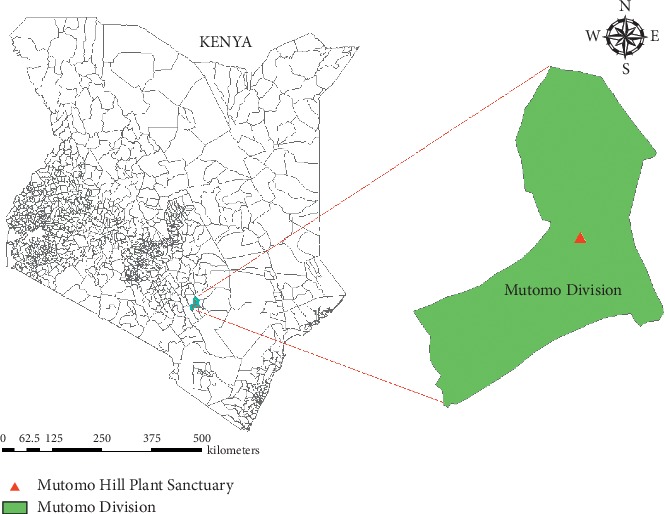
Map of Kenya showing the location of Mutomo division and Mutomo hill plant sanctuary.

The study was carried out in Mutomo hill plant sanctuary and its environs. Mutomo hill plant sanctuary is geographically located within the Mutomo division; hence Mutomo division was selected to represent the environs of Mutomo hill plant sanctuary. Mutomo division is located at 38°12′ East and 1°50′ South in Kitui county (Figure 1). Kitui county is largely a low plateau rising from 300 m above the sea level and interrupted by various inselbergs reaching about 1638 m above the sea level [53] where the highest altitudes reach about 1800 m [54]. Generally, the climate of Kitui county varies from arid to semiarid and is characterized by a minimum mean annual temperature varying from 14°C to 22°C and a maximum mean annual temperature ranging from 26°C to 34°C. There are two rainy seasons where the long rains start from March and end in June while the short rains start in October and end in December with a mean annual rainfall ranging from 250 mm to 1050 mm [54]. The low-lying areas receive low rainfall and are extremely hot. The vegetation of Kitui county is characterized by low, stunted, dense thorn bushes with thick undergrowth and occasional baobab trees. Much of the area lacks forests except on the hills [55] where scrublands and wooded bushlands can be found [56] dominated by *Drypetes*, *Combretum*, *Vepris,* and *Croton* species [46]. The geography of Kitui county is characterized by several hilltops which contain a high diversity of plant and animal species [46]. Such highlands provide a link between the coastal forests and the highland forests, resulting in the survival of unique species adapted to the individual highlands [46, 57]. One of such important highlands is the Mutomo hill plant sanctuary, located 125 miles East of Nairobi. The sanctuary is a ten-hectare piece of land, about 1211 m in length and rising to an altitude of about 1000 m from red lateritic plains to worn granitic gneiss [49, 50]. Its floral diversity is rich and includes succulent and xerophytic plants [49]. The plant diversity of Kitui county is high and is used by the local communities for traditional foods, tisanes, medicines among other uses [53, 57].

Kitui county is inhabited by Kamba people who practice agriculture and semipastoralism [40, 55] which involves growing a variety of crops and keeping livestock [53, 57] where the cattle are kept as a security against famine [58]. Most of the population in the county relies on agriculture for economic income, where the main food crops include maize, millet, sorghum, green grams, beans, peas, cassava, and sweet potatoes [54]. Literature reveals that as early as 1911, the Kitui Kamba traditional way of life was being interrupted by foreign groups including the colonial governments [58]. Despite this, they have conserved their indigenous knowledge on plant utilization and are reportedly one of the ethnic groups in Kenya who have conserved their traditional knowledge on medicinal plants [40, 53].

### 2.2. Ethnobotanical Data Collection and Analysis

The field survey was conducted between July 2018 and February 2019. Prior to the field survey, the herbal practitioners were identified with the help of the local administrative authorities who included the subchiefs of Mutomo division and the rangers at Mutomo hill plant sanctuary. In addition, the herbalists recommended the researchers to other herbalists within the study area. The informants were the herbalists who were practicing traditional medicine at the time of study or those who had reduced working periods due to age or to be involved in other activities such as farming. Interviews were administered in Kamba language, which is the main dialect spoken in the area and later translated into English. Interviews were recorded in semistructured open-ended questionnaires at the homesteads of the herbalists while in some cases, the herbalists met with the researchers at shopping centers or in other appropriate places such as farmlands where some of them collected the medicinal plants. The herbalists first mentioned the medicinal plants they had used and later guided the researchers to the wild where the plants were collected. Interviews with each informant lasted between two to five hours involving field surveys in different ecotypes including hills, forests, and farmlands throughout the six sublocations of Mutomo division and within the Mutomo hill plant sanctuary. Data collected included the demographic information of the informant (name, age, sex, occupation, and level of education) and the botanical information (vernacular name of the plant, its source, parts used, medicinal uses, and methods of drug preparation and administration). For each plant species cited, an herbarium voucher material was collected. Photographs of all the medicinal plants cited were taken to help during identification. Identification was done following the local monographs of the Kenyan flora [59, 60] and the Flora of Tropical East Africa [61]. The voucher collections were later verified at the East African (EA) herbarium in Kenya. All the voucher materials reported in this study were deposited at the EA (the collection details: localities, voucher numbers, elevation, and coordinates at sampling points are summarized in Table 1). The nomenclature of all the species names was further verified using The Plant List (http://www.theplantlist.org/).

The plant parts used for herbal medicines were classified according to Cook [62] while the plant growth habits were classified based on the monographs of Kenyan flora [59, 60]. The health conditions reported in the study area were classified according to Cook [62], where all the diseases reported by the informants belong to level one category of “*medicines*” while each disease cited by the informants belongs to a certain “*level two category*” within “*level one category*.” Where the disorders could not be matched directly to level two categories, the body parts affected by the disorders were matched with their level two respective categories until all the ailments reported were categorized. The data were entered into an excel spreadsheet and analyzed to determine plant growth habit proportions, proportions of plant parts used, diseases treated, and the priority plant species based on Relative Frequency of Citation values (RFC). The RFC for each species was quantitatively calculated using the formula RFC = (FC/*N*)(0 < RFC < 1) [63], where the FC is the Frequency Citation, while *N* is the number of respondents participating in a particular study (here, *N* = 48). The RFC determines the consensus between the informants on the use of a reported plant species in a given area which in turn gives its local importance. The RFC values range from 0 to 1, where RFC = 0 indicates that no informant mentioned the particular plant species while RFC = 1 indicates that the plant species was cited by all informants in a particular study.

## 3. Results

### 3.1. Sociodemographic Data

A total of 48 local herbalists composed of 32 males and 16 females, aged between 32 and 96 years, with an average age of 68.25 years were interviewed. One herbalist was old (96 years) and collected the medicinal plants near his homestead. Two of the female herbalists sold the medicinal plants at Mutomo market, while all the other herbalists were farmers and served their customers or patients when consulted or visited. A majority (27%) of the herbalists were aged between 71 and 80 years and between 61 and 70 years (25%) while a number of them (18.75%) were over 80 years old. Only 2.08% of the herbalists were below 40 years old. In terms of education, majority of the herbalists (77.08%) had no formal education, 20.83% had at least attended primary school education although none of them had completed, while only 2.08% of the herbalists had completed secondary school training (Table 2).

### 3.2. Medicinal Flora

**Figure 2:**
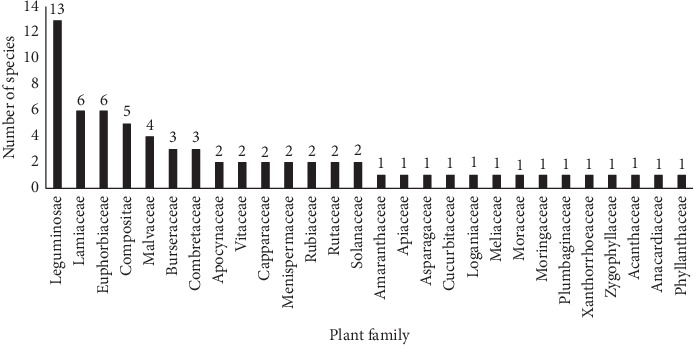
Distribution of plant species in different families.

Sixty-eight (68) plant species distributed in 28 families and 54 genera (Table 3) were reported to be used in the management of various human health conditions. Leguminosae comprised the majority of the species (13), followed by Euphorbiaceae and Lamiaceae, each with six species. A majority of the plant families comprised one species (Figure 2).

### 3.3. Growth Habit of the Medicinal Plants

**Figure 3:**
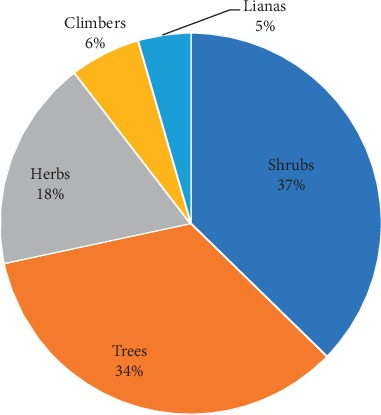
Distribution of plant growth habits.

Shrubs (37%) and trees (34%) were cited by the informants to be frequently collected for herbal medicine while herbs (18%), climbers (6%), and lianas (4%) were least cited (Figure 3).

### 3.4. Plant Parts Collected for Herbal Medicine

Roots and barks (each with 19 species) were reported to be the common plant parts collected for herbal medicine, followed by leaves (16 species), exudates (11 species), aerial parts (9 species), whole plants (4 species), fruits (4 species), stems (2 species) and inflorescence (1 species).

### 3.5. Frequently Collected Medicinal Plants

**Figure 4:**
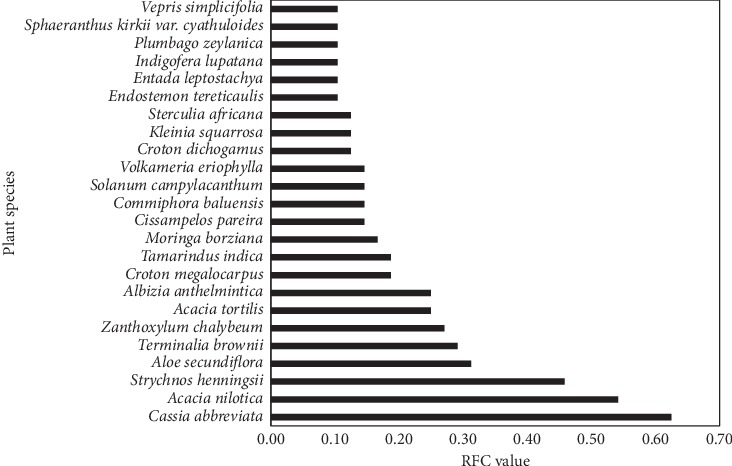
Frequently used plant species.

The RFC for each plant species was calculated. A summary of the RFC values of all medicinal plants reported from Mutomo hill plant sanctuary and its environs are outlined (Table 3). Plant species with five or more citations (RFC ≥ 0.10) were considered as the priority plant species. In total, 24 plant species were frequently cited by the respondents. *Cassia abbreviata* (RFC = 0.63), *Acacia nilotica* (RFC = 0.54), *Strychnos henningsii* (RFC = 0.46), and *Aloe secundiflora* (RFC = 0.31) had the highest RFC values (Figure 4).

### 3.6. Health Conditions Reported

In total, 13 disease categories including ethnoveterinary diseases were reported during the study. Infectious diseases and infestations were found to be treated with majority of the plant species (33) followed by digestive system disorders (24 plant species) (Table 4). These two disease categories are discussed in detail due to their perceived important role in the health of the people in the study area.

## 4. Discussion

### 4.1. Sociodemographic Data

All the herbalists who participated in the study were over 30 years old, mostly dwelling in the rural areas. People living in the rural areas are reported to acquire more ethnobotanical knowledge probably due to the availability of plants in their surroundings [75]. A large percentage of the herbalists had no formal education, a proportion that was below the national average level of adult literacy in Kenya [76]. The low level of formal education in herbal practitioners has also been reported in other regions of Kenya [67, 77]. This contradicts a previous study in Kitui county where most of the respondents had acquired formal education up to primary school level [34]. According to a report by the county government of Kitui, people over 65 years old are considered aged and not economically productive [54]. In this study, however, the aged people were found to be actively practicing traditional medicine which in some instances was a source of income since some of them sold the herbal products in the market. Elderly people practicing traditional medicine has been reported elsewhere in Kenya [77]. It is also argued that the level of ethnobotanical knowledge may increase as people advance in age probably due to increased responsibilities [75] which partly explains the high number of aged herbalists. Generally, the population of females is higher than that of males in Kitui county [54], yet most of the herbalists in the study were males. This agrees with Kaingu et al. [77] who reported males to comprise most of the herbalists in a previous study, although this is contradicted by a recent study in Kitui county [34] where women were reported to comprise most of the respondents. Despite such variations, there may be no difference in the level of ethnobotanical knowledge between the two genders [75].

Most of the herbalists preferred collecting medicinal plants on their own, which in some cases involved travelling for hours, unless a plant species could be found in their vicinity or was an obvious tree, where they could ask for assistance. The herbalists who stored herbal materials for future use also kept it a secret and normally stored them mixed together or ground into a powder to make utilization or identification by an unfamiliar person difficult. In addition, most of the herbalists chose to be interviewed in the absence of other people including some of their family members except their spouses and one or two confidants. This is likely to contribute to the degradation of traditional knowledge in young generations since the chances of acquiring it are controlled by those who possess it. Although Kisangau et al. [34] found that most of the respondents had acquired traditional knowledge through apprenticeship from family members, Muthee et al. [67] argues that the poor traditional knowledge in young generations could be an indication that it is not being transferred or is acquired after a long period of time. This is further supported by Nanyingi et al. [36] who found that the knowledge on the treatment of some diseases was possessed by the elder members of the society. There are reports that in some regions of Kenya, the young generations are ignorant of traditional practices after taking up formal education [37, 45]. However, external factors such as education may not negatively affect the sustainability of traditional knowledge in people who have already acquired it [75]. It is likely that there exists a barrier limiting the transfer of traditional knowledge between the old and the young generations, a complexity that needs elaboration in future studies.

### 4.2. Medicinal Flora

Leguminosae was the dominant plant family reported during the study followed by Euphorbiaceae and Lamiaceae. Leguminosae was also reported to be among the frequently used plant families in treatment of malaria in Kwale community [78] and in the management of HIV/AIDS in Mfangano Island in Kenya [15]. In the study of medicinal plants used by the Marakwet community, Mimosaceae was the dominant plant family reported [79] while in the study of medicinal plants of Tana river county, Euphorbiaceae, Leguminosae, and Lamiaceae were found to be among the frequently used plant families [30, 77]. In the flora of Kitui county, Leguminosae is the largest plant family [46]. It is also the largest plant family in the flora of Kenya [24]. African legumes are tolerant to drought and therefore comprise important resources for people living in arid areas [80]. Considering the amount of rainfall received in drylands of Kenya [81] and its elevational range [25], Kitui county can be considered as a typical dryland region; hence the large number of legumes could be attributed to the arid conditions of the area.

Shrubs and trees were the dominant life forms reported, which agrees with the results of another study in Kenya [36]. An ethnobotanical study among the Marakwet community in Kenya reported trees to be the frequently used plant life forms [79] while among the Kwale people of Kenya, shrubs were found to be frequently utilized for the treatment of malaria [78]. In mount Elgon, a study of medicinal plants reported that trees were the dominant life forms used followed by shrubs [41]. Generally, the vegetation of the study area is dominated mostly by dry forests composed of shrubs [46, 56, 64]. Shrubs can also be obtained from many areas including bushlands and farmlands [44], making it easier for the local communities to access them.

In this study, roots and barks were found to be frequently collected for herbal medicine. A single plant species may have several parts collected for herbal medicine [82] which was also reported during the study. Some plant species were reported to be used as whole plants. The use of the whole plants in Kenya is reported to be applied in cases of small plants, herbaceous plants, or epiphytes [44]. In a similarly arid region in Kenya, roots and barks were found to be frequently collected for herbal medicine [45]. In a study of medicinal plants used by the Sabaot people of mount Elgon, roots were found to be frequently utilized followed by the barks and leaves [41]. Moreover, in a survey of medicinal plants in the urban areas of Kenya, roots and barks were found to be the common plant parts sold [21]. In some studies, leaves are reported to be frequently collected with significant proportions of barks and roots being reported [34, 82, 83]. According to Malonza et al. [46], debarking and uprooting are some of the common techniques of harvesting medicinal plants used by the people of Kitui. Harvesting of root and bark increases the level of vulnerability of plants since the two parts take a longer time to regenerate when compared to the leaves; hence the chances of survival of debarked trees are rare [34, 47]. Debarked plants are likely to be in danger of overexploitation; hence their conservation status needs further investigation [82]. Since plants found in dry areas are perceived to be of better medicinal quality, they are preferred for collection by traders and herbalists, thus risking degradation due to their high demand [84]. In the semiarid areas of Kenya, the effects of overutilization, desertification, and global warming have subjected biodiversity to increased pressure [45]. A combination of such factors with the vulnerable nature of semiarid lands may lead to further reduction of habitats which harbor medicinal plants [67]. Therefore, substituting the roots and barks with plant parts such as leaves and aerial parts may reduce the pressure exerted on medicinal plants [47]. In addition, bioactive compounds from plants are mostly extracted using water [41, 45] whose efficacy may be low compared to other media such as methanol, suggesting that ineffective media for extracting plant-based compounds may lead to wastage of the harvested materials; hence traditional healers need to be trained on other potential effective methods [45]. Conservation initiatives of the plant species endangered by overexploitation are therefore necessary [46] and Mutomo hill plant sanctuary is potentially a suitable area for such initiatives in Kitui county.

There were 24 plant species frequently collected by the herbalists in the study area, all of which can be treated as priority plants based on Njoroge et al. [40]. From this study, five plant species (*Zanthoxylum chalybeum, Terminalia brownii, Croton megalocarpus*, *Albizia anthelmintica,* and *Aloe secundiflora*) have been reported as priority medicinal plants elsewhere in Kitui county [40]. *Zanthoxylum* and *Strychnos* species are also among the most preferred plant species for herbal medicine in Kitui county [46]. *Acacia nilotica* and *Strychnos henningsii* were also found to be among the most popular plant species used in the management of respiratory infections at Kibwezi, an area adjacent to Mutomo subcounty [45]. As reported in a previous study [41], most of the plant species were obtained from the wild. In this study, all the *Commiphora* species were found to be cultivated by some informants as hedge plants especially along fences. This is because *Commiphora* species can be propagated easily from cuttings since they root easily once driven into the ground [60]. Cultivation of plants as live fences has been undertaken by some local communities in Kenya as a way of preserving scarce medicinal plants [36]. Other plant species cultivated were *Aloe secundiflora*, *Croton megalocarpus,* and *Melia volkensii*. The herbalists reported an increasing scarcity of *Strychnos henningsii* and *Vepris simplicifolia*, relating it to land conversion for farmlands and private development. The decline of *Strychnos henningsii* in the wild was previously reported in Kitui county [40]. Due to the increasing scarcity of medicinal plants, some herbalists reported retaining *Terminalia brownii, Tamarindus indica*, *Balanites aegyptiaca,* and *Moringa borziana* in their farmlands for future medicinal uses. Njoroge et al. [40] also reported that *Terminalia brownii*, *Croton megalocarpus,* and *Aloe secundiflora* were preserved in the farmlands for future medicinal uses. The abundance of useful plants has been reported to decline as a result of fluctuating rainfall patterns and anthropogenic activities such as selective harvesting, overexploitation, and expansion of farmlands [85]. In Kitui county, *Acacia tortilis* and *Terminalia brownii* are also reported to be used for charcoal [34] which may further reduce their abundance in the wild. Some medicinal plants found in this study such as *Zanthoxylum chalybeum* and *Albizia anthelmintica* are also sold in the urban areas of Kenya [21]. During the study, *Cassia abbreviata*, *Terminalia brownii,* and *Vepris simplicifolia* were found to be sold in Mutomo market. Several important collection sites for medicinal plants in Kenya are reported to have been converted into farmlands [86]. In an ethnobotanical study in Zanzibar, the abundance of some medicinal plants is reported to have decreased making it difficult to obtain them from the wild [87]. The supply of medicinal plant products sold in the Kenyan urban centers is also decreasing [21]. Amir et al. [88] reported that few people in Tanzania cultivated medicinal *Aloe* species and that most of them were being obtained from the wild, resulting in the decline of wild *Aloe* populations. *Aloe* species in Kenya, are however threatened by factors related to the increase in human and livestock populations and international trade rather than utilization for herbal medicinal [89]. Generally, medicinal plants in Kenya are threatened by both natural and anthropogenic factors where the majority of them have declined due to deforestation [36].

### 4.3. Medicinal Uses

The medicinal applications of most of the plant species reported in this study have been reported before although the diseases treated, the parts used, the methods of preparation, and administration of the drugs may differ in some instances. A previous study reported on novel therapeutic applications of some plant species in Kenya [41]. However, further botanical investigations of voucher collections stored in various herbaria are necessary before conclusions can be arrived at. Plant species belonging to the same genus may be used for the management of similar health conditions. Such species may have the same local names such as those of genus *Sphaeranthus*, which are reported to be used in the treatment of malaria and edema [64]. Morgan [65] also found out that the same local names were being applied by the local community to refer to *Indigofera* and *Crotalaria* species. During the field survey, an herbalist mentioned that *Indigofera arrecta* and *I. lupatana* could at times be confused since the two plants share the same local name in the study area. Substituting *I. arrecta* with *I. lupatana* was sometimes applicable although the latter was reported to be more effective. Phytochemical investigations to determine the effectiveness of the two species in the management of the said ailments are therefore in need. Some cases of health management involved the treatment of symptoms of the reported ailments. For example, reduced appetite was reported to be a symptom of a swollen diaphragm; hence the two conditions were managed simultaneously. Some cases of herbal preparations involved mixing of different plant species, which the herbalists claimed was a remedy for notorious diseases or a suspected combination of two diseases. Mixing of medicinal plants for the treatment of a single ailment has been reported in other studies [32, 36]. In addition, some herbal products sold in Kenyan urban areas for the treatment of some diseases are prepared mixed [21]. Since the efficacies of most plant species used in traditional medicine have not been tested, their role in disease management cannot be ascertained [45]. Ethnobotanical studies in Kenya are also considered few in light of the high plant diversity in the country [36]. Although literature reveals that an enormous data on medicinal plants of Kenya exist [1], ethnomedicinal applications of some plant species have not been documented to date. As a result, Njoroge et al. [40] emphasized the need for the documentation of ethnomedicinal uses of regional floras.

Infections and digestive system disorders are treated with most of the medicinal plant species in some parts of Kenya [32, 34]. Tuberculosis was reported to be managed by chewing the leaves of *Combretum hereroense* and swallowing the extract. Tuberculosis is a bacterial disease majorly affecting the lungs although it has been reported to advance to the ears and the tonsils especially in Africa [90–92]. Earache and tonsillitis were also reported to be managed using herbal medicines. However, based on this ethnobotanical survey, the relationship between tuberculosis and such ailments cannot be ascertained. *Acacia nilotica*, *A. tortilis*, *Strychnos henningsii,* and *Cissus aphyllantha* were reported to be used for the treatment of pneumonia. *Acacia nilotica* and *Strychnos henningsii* have been found to show efficacy against the pathogens causing respiratory infections including pneumonia [45]. Pneumonia is reportedly the leading disease in childhood mortality, especially in marginalized communities of developing countries [93]. In recent years, respiratory infections have become resistant to antibiotics, resulting in ineffectiveness in their treatment which has led to advocacy in search of plant-based antimicrobial drugs [45, 82, 94]. Decoctions of *Lannea schweinfurthii* and *Plumbago zeylanica* were reported for the treatment of gonorrhea. A similar application of *Lannea schweinfurthii* has been reported before [64]. One herbalist admitted to advising his family members to take a decoction of *Lannea schweinfurthii* against gonorrhea at least once a month to avoid the chances of contracting the disease. It is likely that the local herbalists are aware of the adverse effects associated with the disease especially in women in spite of its asymptomatic nature [95]. Gonorrhea pathogen is reported to show resistance to clinical therapies [96]; hence medicinal plants may offer other opportunities for the development of novel drugs against such infections.

Some herbalists reported that pain in joints, general body pains, and headache were symptoms of malaria. These conditions together with malaria were therefore managed by the herbalists using the same medicinal plants. Although such ailments are categorized under muscular-skeletal disorders, their association with malaria has been reported in other studies in Kenya [97, 98]. *Cassia abbreviata* and *Albizia anthelmintica* which were reported for the treatment of malaria have been reported to have no or a weak antiplasmodial activity; hence they are likely to be applied in the treatment of conditions that accompany malaria [99]. Plant species such as *Strychnos henningsii*, *Aloe secundiflora*, and *Zanthoxylum chalybeum* which have been reported for the treatment of chronic joint pains [100] were reported for treatment of malaria during the study. A bath from an infusion of *Kleinia squarrosa* was also reported to be used against malaria. Chemical analysis of *Kleinia squarrosa* has revealed that essential oils from the plant contain volatile compounds that act as mosquito repellants [101] which further supports its application in the management of mosquito-related ailments. Malaria was reported to be treated through the administration of decoctions, infusions, and bathes. Historical treatments of malaria with plant-based derivatives are the use of quinine and artemisinin as antimalarial drugs [97]. Treatment of malaria with medicinal plants is also widely reported in Kenya [17, 98]. Some medicinal plants which were reported for treatment of malaria such as *Vepris simplicifolia* and *Zanthoxylum chalybeum* have proven antiplasmodial activities [99]. Malaria is a major hindrance to economic growth in Sub-Saharan Africa [102] and it is also among the leading diseases in childhood mortality and in deaths of pregnant mothers in Africa [93]. In recent years, it has become difficult to treat malaria owing to the development of plasmodial resistance against the available clinical drugs [103]. The high prevalence of malaria infection in rural areas of Kenya has led the local communities to rely on medicinal plants, with some rural inhabitants reporting them to be more effective and cheaper compared to clinical therapies [98].

Viral infections reported during the study to be managed using medicinal plants included yellow fever, measles, smallpox, colds, and flu. Common colds are caused by numerous viruses belonging to different families and they display varying clinical manifestations. Influenzas, the viruses causing flu are among the viruses associated with common colds. A universal mode of treatment and prevention of the disorders arising from common colds has not been developed due to the varying pathogenic mechanisms of the viruses [104]. Yellow fever was reported to be treated through bathing an infusion of *Terminalia brownii*, as previously reported [34, 60]. Yellow fever is a disease in tropical South America and Africa with no specific treatment and is currently managed using vaccines [105, 106]. *Tamarindus indica* was reported for the treatment of measles and smallpox by consumption of the fruits, steam bathing, or by taking an infusion from the fruit and leaves, as reported elsewhere [64]. In addition, a bath from an infusion of *Kleinia squarrosa* was also reported to treat smallpox and measles. According to two herbalists, cases of smallpox outbreak are nowadays rare. Vaccinations have effectively dealt with smallpox and are on the verge of eliminating measles, while the vaccine against yellow fever virus induces a long-term immunity once administered [107]. Other than vaccination, measles is prevented through supplementation of vitamin A [108, 109]. However, Sub-Saharan Africa is classified by the WHO as a region with the highest rates of vitamin A deficiency [109]. Although control of some viral infections through vaccination has been effective, viruses still develop resistance to drugs making the treatment of associated diseases extremely difficult [110]. Hence the role of medicinal plants in the treatment of viral diseases cannot be neglected.

*Taenia* worms were reported to be primarily expelled using a decoction of *Albizia anthelmintica*, an application that has been quoted in various studies [1, 34, 60, 64]. The plant is also reported to be antimicrobial [31]. In addition, an infusion of *Commiphora edulis* was reported to be used against tapeworms. Pinworm infestation was reported to be managed through exposure to smoke from burning seeds of *Ricinus communis*, an application that is likely to be facilitated by the poisonous nature of the seeds [1, 66]. Ringworms were reported to be treated with topical applications of exudate from *Edithcolea grandis* and the fruit juice of *Solanum campylacanthum*. Ringworms are fungal infections [62], locally referred to as “*masilingi*” by the local herbalists and infecting the surface of the skin. *Solanum campylacanthum* is reported to have an antifungal activity [111] supporting its ethnobotanical application in the treatment of ringworms. Skin ailments are mostly treated through direct topical application of herbal preparations [38] although a decoction of *Terminalia prunioides* was also reported to be drunk against ringworms.

Gastrointestinal problems are common health problems in rural areas of Kenya where in some ethnobotanical studies, they have been found to be managed with most of the medicinal plants surveyed [32, 86]. According to the WHO, diarrhea is one of the world's leading killer diseases in children below the age of five [93]. The herbalists associated its outbreak with poor sanitary conditions especially dirty drinking water, which is also a major cause of diarrhea according to the United Nations Children's Fund [109]. Diarrhea was reported to be associated with stomach-ache and constipation as also reported elsewhere [42]. Those ailments were reported by the herbalists to be mostly managed using herbal infusions and decoctions. Some plant species reported for the treatment of gastrointestinal ailments such as *Croton megalocarpus*, *Terminalia brownii,* and *Cissampelos pareira* have been reported to have antimicrobial activities [31, 112, 113]. On two different accounts, the herbalists reported to advising pregnant mothers against taking infusions of *Solanum campylacanthum* and *Cissampelos pareira* for stomach problems as they might induce abortion. However, *Solanum campylacanthum* is reported to play important roles during labor and in maintaining pregnancy [13]. Although medicinal plants may have profound effects on pregnant mothers [114], they also play important roles in the development of fetus and in the health of the mothers [14]. Side effects of medicinal plants may result from improper dosage and the effects may be nonfatal [98]. Peptic ulcers were reported to be treated using an infusion of *Aloe secundiflora* or a decoction of *Terminalia prunioides*. *Aloe secundiflora* is reported to have an antimicrobial activity [115] which further supports its ethnobotanical application. Toothache was reported to be managed using *Solanum campylacanthum*, *Zanthoxylum chalybeum,* and *Ficus sycomorus* where the first two species are reported to have antimicrobial activities [111, 116]. Silver nanoparticles synthesized from the latex and leaves of *Ficus sycomorus* have been found to have a high antibacterial inhibition, proving that the plant has a potential in the treatment of bacterial infections [117]. However, according to one herbalist, the use of *Ficus sycomorus* latex for toothache is dangerous since it might spread in the mouth resulting in the loss of other teeth through corrosion which in turn leads to cracking and splitting of teeth into smaller pieces which fall with time.

Historically, the Kamba people of Kitui were semipastoralists [55, 58] and this culture has persisted to date [53]. Although the present study focused on documentation of medicinal plants used in the management of human diseases, the herbalists reported some plant species used for the management of livestock diseases (Table 5). Future studies are necessary to determine the diversity of plants used for the management of livestock health conditions in the study area and in Kitui county at large.

## 5. Conclusion and Recommendations

This documentation contributes to safeguarding the indigenous knowledge of medicinal plants in the study area, which might be useful for the future conservation of such plants in the sanctuary. Efficacy studies of the plant species reported, especially the priority plants are necessary to determine their potential in the development of novel drugs. Further studies are necessary to determine the diversity of plant species sold in the urban areas of Kitui, which might be useful for conservation considerations. The herbalists in the study area need capacity building to develop home gardens for the cultivation of the medicinal plants so as to ease the pressure exerted on wild populations. In addition, government institutions involved in biodiversity conservation in Kenya and the county government need to extend their conservation efforts to the Mutomo hill plant sanctuary. Further studies to determine the suitability of the sanctuary for the conservation of medicinal plants are necessary.

## Figures and Tables

**Table 1:** A summary of the collection details of the voucher materials cited by the herbalists at Mutomo hill plant sanctuary and its environs.

Family and species name	Voucher number	Area of collection	Elevation and coordinates
Acanthaceae			
*Barleria eranthemoides*	Mutie MU0215	Kitoo sublocation, Ndiini village	836 m, 1°55′20.4″ S, 38°07′11.2″ E
Amaranthaceae			
*Achyranthes aspera* var. *sicula*	Mutie MU0192	Kawelu sublocation, Ngomeni village	713 m, 1°49′49.8″ S, 38°12′23.0″ E
Anacardiaceae			
*Lannea schweinfurthii*	Mutie MU0271	Kibwea sublocation	882 m, 1°52′56.1″ S, 38°15′14.6″ E
Apiaceae			
*Steganotaenia araliacea*	Mutie MU0243	Mutomo hill plant sanctuary	910 m, 1°49′49.8″ S, 38°12′23.0″ E
Apocynaceae			
*Calotropis procera*	Mutie MU0238	Kibwea sublocation	882 m, 1°52′56.1″ S, 38°15′14.6″ E
*Edithcolea grandis*	Mutie MU0216	Kitoo sublocation, Ndiini village	836 m, 1°55′20.4″ S, 38°07′11.2″ E
Asparagaceae			
*Sansevieria perrotii*	Mutie MU0063	Mutomo hill plant sanctuary	910 m, 1°49′49.8″ S, 38°12′23.0″ E
Burseraceae			
*Commiphora baluensis*	Mutie MU0190	Kawelu sublocation	713 m, 1°49′49.8″ S, 38°12′23.0″ E
*Commiphora edulis*	Mutie MU0193	Kawelu sublocation	713 m, 1°49′49.8″ S, 38°12′23.0″ E
*Commiphora habessinica*	Mutie MU0174	Kitoo sublocation	836 m, 1°54′46.1″ S, 38°11′00.9″ E
Capparaceae			
*Boscia coriacea*	Mutie MU0222	Kawelu sublocation	705 m, 1°47′12.3″ S, 38°14′19.5″ E
*Maerua endlichii*	Mutie MU0201	Kibwea sublocation	705 m, 1°47′12.3″ S, 38°14′19.5″ E
Combretaceae			
*Combretum hereroense*	Mutie MU0203	Kibwea sublocation	676 m, 1°44′09.9″ S, 38°13′36.0″ E
*Terminalia brownii*	Mutie MU0226	Kibwea sublocation	882 m, 1°52′56.1″ S, 38°15′14.6″ E
*Terminalia prunioides*	Mutie MU0091	Mutomo hill plant sanctuary	910 m, 1°49′49.8″ S, 38°12′23.0″ E
Compositae			
*Aspilia pluriseta*	Mutie MU0225	Kibwea sublocation	882 m, 1°52′56.1″ S, 38°15′14.6″ E
*Kleinia squarrosa*	Mutie MU0199	Kawelu sublocation	713 m, 1°49′49.8″ S, 38°12′23.0″ E
*Launaea cornuta*	Mutie MU0209	Kitoo sublocation	836 m, 1°54′46.1″ S, 38°11′00.9″ E
*Sphaeranthus kirkii* var. *cyathuloides*	Mutie MU0219	Kawelu sublocation	853 m, 1°51′03.3″ S, 38°09′57.8″ E
*Tridax procumbens*	Mutie MU0227	Mwala sublocation	868 m, 1°52′56.2″ S, 38°15′14.2″ E
Cucurbitaceae			
*Kedrostis pseudogijef*	Mutie MU0212	Kitoo sublocation, Ndiini village	836 m, 1°55′20.4″ S, 38°07′11.2″ E
Euphorbiaceae			
*Croton dichogamus*	Mutie MU0245	Kitoo sublocation, Ndiini village	836 m, 1°55″20.4′ S, 38°07″11.2′ E
*Croton megalocarpus*	Mutie MU0228	Mwala sublocation	868 m, 1°52′56.2″ S, 38°15′14.2″ E
*Euphorbia crotonoides*	Mutie MU0009	Mutomo hill plant sanctuary	910 m, 1°49′49.8″ S, 38°12′23.0″ E
*Euphorbia scheffleri*	Mutie MU0038	Mutomo hill plant sanctuary	910 m, 1°49′49.8″ S, 38°12′23.0″ E
*Euphorbia uhligiana*	Mutie MU0066	Mutomo hill plant sanctuary	910 m, 1°49′49.8″ S, 38°12′23.0″ E
*Ricinus communis*	Mutie MU0205	Kitoo sublocation	836 m, 1°54′46.1″ S, 38°11′00.9″ E
Lamiaceae			
*Endostemon tereticaulis*	Mutie MU0231	Kandae sublocation	868 m, 1°52′56.2″ S, 38°15′14.2″ E
*Hoslundia opposita*	Mutie MU0244	Mutomo hill plant sanctuary	910 m, 1°49′49.8″ S, 38°12′23.0″ E
*Plectranthus lasianthus*	Mutie MU0217	Kitoo sublocation, Ndiini village	836 m, 1°55′20.4″ S, 38°07′11.2″ E
*Plectranthus otostegioides*	Mutie MU0235	Kibwea sublocation	882 m, 1°52′56.1″ S, 38°15′14.6″ E
*Pycnostachys umbrosa*	Mutie MU0213	Kitoo sublocation, Ndiini village	836 m, 1°55′20.4″ S, 38°07′11.2″ E
*Volkameria eriophylla*	Mutie MU0291	Mwala sublocation	868 m, 1°52′56.2″ S, 38°15′14.2″ E
Leguminosae			
*Acacia brevispica*	Mutie MU0031	Mutomo hill plant sanctuary	910 m, 1°49′49.8″ S, 38°12′23.0″ E
*Acacia mellifera*	Mutie MU0246	Mutomo hill plant sanctuary	910 m, 1°49′49.8″ S, 38°12′23.0″ E
*Acacia nilotica*	Mutie MU0224	Kibwea sublocation	882 m, 1°52′56.1″ S, 38°15′14.6″ E
*Acacia tortilis*	Mutie MU0198	Kawelu sublocation	713 m, 1°49′49.8″ S, 38°12′23.0″ E
*Albizia anthelmintica*	Mutie MU0194	Kawelu sublocation	713 m, 1°49′49.8″ S, 38°12′23.0″ E
*Cassia abbreviata*	Mutie MU0221	Kawelu sublocation	853 m, 1°51′03.3″ S, 38°09′57.8″ E
*Delonix elata*	Mutie MU0233	Kibwea sublocation	868 m, 1°52′56.1″ S, 38°15′14.6″ E
*Dichrostachys cinerea*	Mutie MU0232	Kandae sublocation	868 m, 1°52′56.2″ S, 38°15′14.2″ E
*Entada leptostachya*	Mutie MU0229	Mwala sublocation	868 m, 1°52′56.2″ S, 38°15′14.2″ E
*Indigofera lupatana*	Mutie MU0053	Mutomo hill plant sanctuary	910 m, 1°49′49.8″ S, 38°12′23.0″ E
*Senna occidentalis*	Mutie MU0237	Kibwea sublocation	882 m, 1°52′56.1″ S, 38°15′14.6″ E
*Tamarindus indica*	Mutie MU0208	Kitoo sublocation	836 m, 1°54′46.1″ S, 38°11′00.9″ E
*Tephrosia villosa*	Mutie MU0054	Mutomo hill plant sanctuary	910 m, 1°49′49.8″ S, 38°12′23.0″ E
Loganiaceae			
*Strychnos henningsii*	Mutie MU0200	Mwala sublocation	868 m, 1°52′56.2″ S, 38°15′14.2″ E
Malvaceae			
*Grewia tembensis*	Mutie MU0242	Mutomo hill plant sanctuary	910 m, 1°49′49.8″ S, 38°12′23.0″ E
*Grewia tephrodermis*	Mutie MU0220	Kawelu sublocation	853 m, 1°51′03.3″ S, 38°09′57.8″ E
*Grewia villosa*	Mutie MU0206	Kitoo sublocation	836 m, 1°54′46.1″ S, 38°11′00.9″ E
*Sterculia africana*	Mutie MU0211	Kitoo sublocation	836 m, 1°54′46.1″ S, 38°11′00.9″ E
Meliaceae			
*Melia volkensii*	Mutie MU0223	Kibwea sublocation	882 m, 1°52′56.1″ S, 38°15′14.6″ E
Menispermaceae			
*Chasmanthera dependens*	Mutie MU0039	Mutomo hill plant sanctuary	910 m, 1°49′49.8″ S, 38°12′23.0″ E
*Cissampelos pareira*	Mutie MU0005	Mutomo hill plant sanctuary	910 m, 1°49′49.8″ S, 38°12′23.0″ E
Moraceae			
*Ficus sycomorus*	Mutie MU0202	Kibwea sublocation, Masaa river	676 m, 1°44′09.9″ S, 38°13′36.0″ E
Moringaceae			
*Moringa borziana*	Mutie MU0236	Kibwea sublocation	882 m, 1°52′56.1″ S, 38°15′14.6″ E
Phyllanthaceae			
*Bridelia taitensis*	Mutie MU0239	Mutomo hill plant sanctuary	882 m, 1°52′56.1″ S, 38°15′14.6″ E
Plumbaginaceae			
*Plumbago zeylanica*	Mutie MU0189	Kawelu sublocation, Ngomeni village	713 m, 1°49′49.8″ S, 38°12′23.0″ E
Rubiaceae			
*Hymenodictyon parvifolium*	Mutie MU0050	Mutomo hill plant sanctuary	910 m, 1°49′49.8″ S, 38°12′23.0″ E
*Tennantia sennii*	Mutie MU0027	Kitoo sublocation	836 m, 1°54′46.1″ S, 38°11′00.9″ E
Rutaceae			
*Vepris simplicifolia*	Mutie MU0234	Mutomo hill plant sanctuary	882 m, 1°52′56.1″ S, 38°15′14.6″ E
*Zanthoxylum chalybeum*	Mutie MU0317	Kandae sublocation	868 m, 1°52′56.2″ S, 38°15′14.2″ E
Solanaceae			
*Solanum campylacanthum*	Mutie MU0218	Kitoo sublocation, Ndiini village	836 m, 1°55′20.4″ S, 38°07′11.2″ E
*Solanum tettense*	Mutie MU0214	Kitoo sublocation, Ndiini village	836 m, 1°55′20.4″ S, 38°07′11.2″ E
Vitaceae			
*Cissus aphyllantha*	Mutie MU0247	Mutomo hill plant sanctuary	910 m, 1°49′49.8″ S, 38°12′23.0″ E
*Cissus quadrangularis*	Mutie MU0054	Mutomo hill plant sanctuary	910 m, 1°49′49.8″ S, 38°12′23.0″ E
*Cissus rotundifolia*	Mutie MU0128	Mutomo hill plant sanctuary	910 m, 1°49′49.8″ S, 38°12′23.0″ E
Xanthorrhoeaceae			
*Aloe secundiflora*	Mutie MU0191	Kawelu sublocation, Ngomeni village	713 m, 1°49′49.8″ S, 38°12′23.0″ E
Zygophyllaceae			
*Balanites aegyptiaca*	Mutie MU0210	Kitoo sublocation	836 m, 1°54′46.1″ S, 38°11′00.9″ E

**Table 2:** Demographic information of the herbalists.

Parameter	Percentage (%) composition
*Demographic data*	
Male	66.67
Female	33.33
*Age group in years*	
31–40	2.08
41–50	12.5
51–60	14.58
61–70	25
71–80	27
＞80	18.75
*Level of education*	
Secondary school education	2.08
Primary school education	20.83
No formal education	77.08

**Table 3:** An account of the medicinal plants reported in Mutomo hill plant sanctuary and its environs. The relevant reported medicinal uses of the plant species or literature reporting other medicinal applications of the plant species have been indicated where possible.

Family and species name	Local name	Habit	RFC	Parts collected	Drug preparation and administration	Disease treated	Relevant reported disease treated or use
Acanthaceae							
*Barleria eranthemoides* R.Br. Ex C.B.Clarke	Thangila	Shrub	0.06	Leaf, root	A poultice is applied topically	Foreign objects pierced into the skin and boils	References [13, 64]
Amaranthaceae							
*Achyranthes aspera* var. *sicula L*.	Musekele	Herb	0.02	Whole plant	Burnt to charcoal and ground into a powder which is taken in hot drinks. The powder is alternatively rubbed on a bleeding part of the body that is cut with a razor adjacent to the kidney	Kidney pains	Urinary tract problems [41]
Anacardiaceae							
*Lannea schweinfurthii* Engl.	Kyuasi	Tree	0.02	Bark	An infusion is drunk	Gonorrhea	Gonorrhea, venereal diseases, and gynecological problems [1, 44, 64]
Apiaceae							
*Steganotaenia araliacea* Hochst.	Kivwavui	Tree	0.04	Exudate, aerial parts	An exudate is dropped into the eyes in case of sensitiveness or itching. An infusion from aerial parts is steam bathed for edema	Itching eyes, sensitive eyes, edema	Edema, partial blindness, and body swellings resulting from allergy [1, 41, 64]
Apocynaceae							
*Calotropis procera* (Aiton) Dryand.	Ilumbu	Shrub	0.04	Exudate	Applied topically	Foreign objects pierced into the body	Removing splinters pierced into the body [64]
*Edithcolea grandis* N.E.Br.	Mutulya-ndu	Herb	0.02	Exudate	Applied topically on infected skin	Ringworms	Reference [64]
Asparagaceae							
*Sansevieria perrotti* Warb.	Kiwa kya ndui	Herb	0.02	Exudate	The leaf is heated in hot ash and the exudate squeezed into the ear. The treatment is initiated when the ear starts to ooze pus	Earache	Cuts and body aches [1, 65]
Burseraceae							
*Commiphora baluensis* Engl.	Itula	Tree	0.15	Bark	An infusion is drunk. Alternatively, the bark is dried and ground into a powder which is infused in hot drinks	Diarrhea, swollen diaphragm	Peptic ulcers [64]
*Commiphora edulis* (Klotzsch) Engl.	Kyoa kika	Tree	0.04	Bark	An infusion is drunk	Tapeworms, cough, chest pains	Diarrhea, dysentery, and indigestion [1, 44]
*Commiphora habessinica* (O.Berg) Engl.	Mutungati	Tree	0.06	Exudate	Applied topically	Septic wounds	Old wounds [64]
Capparaceae							
*Boscia coriacea* Graells	Musivu	Shrub	0.06	Leaf	An infusion is drunk	Diarrhea	Stomach-ache [1, 29, 66]
*Maerua endlichii* Gilg and Bened.	Muthitu	Shrub	0.02	Stem	Burnt into charcoal, ground into a powder, and infused in hot drinks	Swollen diaphragm	Reference [29]
Combretaceae							
*Combretum hereroense* Schinz	Mukokola	Shrub	0.06	Leaf	Chewed and the extract swallowed	Tuberculosis, cough	Chest pains [44]
*Terminalia brownii* Fresen.	Kiuku	Tree	0.29	Bark	Bark from the stems or roots is chewed and the extracts swallowed for cough. A warm infusion is bathed for yellow fever and drunk against diarrhea	Cough, yellow fever, and diarrhea	Yellow fever, jaundice, stomach-ache, gastrointestinal complications, and cough [1, 29, 34, 36, 66]
*Terminalia prunioides* M.A.Lawson	Kitoo	Tree	0.02	Bark	A decoction is drunk	Ringworms, peptic ulcers, kidney pains, and aphrodisiac in men	Reference [60]
Compositae							
*Aspilia pluriseta* Schweinf. ex Schweinf.	Muti	Shrub	0.02	Leaf	A poultice is applied topically	Fresh cuts	Wounds and cuts [1, 34, 64, 66]
*Kleinia squarrosa* Cufod.	Mung'endya Nthenge	Herb	0.13	Aerial parts	An infusion is bathed	Malaria, measles, smallpox, and edema	Malaria and edema [13, 34, 64]
*Launaea cornuta* (Hochst. ex Oliv. and Hiern) C.Jeffrey	Uthunga	Herb	0.08	Aerial parts	An infusion is bathed	Malaria, smallpox, edema	Malaria, arthritis, and measles [1, 34, 64]
*Sphaeranthus kirkii* var. *cyathuloides* (O.Hoffm.) Beentje	Musonzoila	Herb	0.10	Aerial parts	An infusion is bathed	Malaria, edema	Headache [1]
*Tridax procumbens* (L.) *L*.	Mumela	Herb	0.04	Leaf	A poultice is applied topically	Fresh cuts, wounds	Wounds [64]
Cucurbitaceae							
*Kedrostis pseudogijef* C. Jeffrey	Mukauw'u	Climber	0.04	Leaf, exudate	A leaf infusion is bathed against measles. An exudate applied on fresh cuts	Measles, fresh cuts	Reference [64]
Euphorbiaceae							
*Croton dichogamus* Pax	Mwalula	Herb	0.13	Bark, leaf	An infusion from the bark is drunk for malaria, back pains, stomach-ache, edema, and cough. An infusion from the leaves is bathed for malaria. A root decoction is drunk for impotence and infertility	Malaria, back pains, stomach-ache, edema, cough, impotence, and infertility	Stomach-ache, chest problems, fever, cough, and as a tonic [1, 60, 66]
*Croton megalocarpus* Hutch.	Muthulu	Tree	0.19	Bark, leaf	An infusion from the bark is drunk for constipation and stomach-ache. The bark is alternatively dried and ground into a powder which is infused in hot drinks. A warm leaf infusion is bathed against malaria and colds	Constipation, stomach-ache, malaria, colds	Stomach-ache, diarrhea, and malaria [1, 36, 64]
*Euphorbia crotonoides* Boiss.	Kamweia	Herb	0.02	Exudate	Applied topically	Warts	Warts [1]
*Euphorbia scheffleri* Pax	Kilembwa	Shrub	0.04	Exudate	Applied topically	Fresh cuts	References [1, 13]
*Euphorbia uhligiana* Pax	Kyaa kinini	Herb	0.02	Whole plant	Burnt to charcoal and ground into a powder which is infused in hot drinks	Hypertension	References [1, 65]
*Ricinus communis L*.	Mbaiki	Herb	0.04	Roots, seeds	An infusion from the root is drunk against diarrhea. The anal opening is exposed to smoke from burning seeds as a remedy for pinworms	Diarrhea, pinworms	Diarrhea and stomach-ache, ruminal impaction or constipation and as a laxative [1, 34, 41, 64, 66, 67]
Lamiaceae							
*Endostemon tereticaulis* (Poir.) M.R.Ashby	Mutaa	Herb	0.10	Aerial parts, roots	An infusion from aerial parts is bathed and drunk against malaria, edema, and diarrhea. Powder from dried roots is infused in hot drinks as a remedy against back pains	Malaria, edema and diarrhea, back pains	Reference [1]
*Hoslundia opposita* Vahl	Musovi	Shrub	0.02	Leaf	An infusion is steam bathed	Edema	Edema [64]
*Plectranthus lasianthus* (Gürke) Vollesen	Kiyo	Herb	0.02	Whole plant	An infusion is steam bathed	Kwashiorkor, edema in children	Reference [68]
*Plectranthus otostegioides* (Gürke) Ryding	Kyeu	Shrub	0.02	Whole plant	An infusion is steam bathed	Kwashiorkor, edema in children	Reference [69]
*Pycnostachys umbrosa* (Vatke) Perkins	Muvou	Shrub	0.02	Root	An infusion is drunk	Diarrhea, stomach-ache	Emetic [60]
*Volkameria eriophylla* (Gürke) Mabb. and Y.W.Yuan	Muumba	Shrub	0.15	Leaf	An infusion is drunk and steam bathed against malaria and edema	Malaria, edema	Edema and malaria [1, 64]
Leguminosae							
*Acacia brevispica* Harms	Mukuswi	Shrub	0.02	Aerial parts	An infusion is steam bathed	Edema	Edema [64]
*Acacia mellifera* (M.Vahl) Benth.	Kithiia	Shrub	0.02	Bark	An infusion is drunk, sometimes mixed with the bark of *Lannea schweinfurthii*	Gonorrhea	References [1, 66]
*Acacia nilotica* (L.) Delile	Kisemei	Tree	0.54	Bark	Chewed and extracts swallowed. An infusion is alternatively drunk	Cough, chest pains, malaria, pneumonia	Malaria, cough, chest pains, and pneumonia [1, 34, 42, 44, 64, 66]
*Acacia tortilis* (Forssk.) Hayne	Mwaa	Tree	0.25	Bark, root	Fresh root is dried in hot ash and smoked like a cigarette as a remedy for flu. The bark is chewed against cough and pneumonia. Alternatively, an infusion from the bark is drunk	Flu, cough, and pneumonia	Cough and colds [29, 64]
*Albizia anthelmintica* Brongn.	Kyoa kisamba	Tree	0.25	Bark	An infusion is steam bathed against edema and drunk against tapeworm, gonorrhea, and measles. The bark is burnt into charcoal and ground into a powder which is applied on septic wounds	Edema, tapeworm, gonorrhea, measles, and wounds	Intestinal worms, edema, old wounds, and gonorrhea [1, 32, 34, 64, 66, 67, 70]
*Cassia abbreviata* Oliv.	Kyalandathe	Tree	0.63	Aerial parts, bark	Aerial parts are chewed and the juice retained in the mouth as a remedy for toothache. A decoction or an infusion from the stem or bark is drunk against cough, malaria, gonorrhea, kidney pains, colds, and pain in joints	Toothache, cough, malaria, gonorrhea, kidney pains, colds, and pain in joints	Gonorrhea, malaria, pneumonia and chest complaints [1, 66]
*Delonix elata* (L.) Gamble	Mwaange	Tree	0.02	Bark	Dried and ground into a powder and topically applied	Septic wounds	Wounds [64].
*Dichrostachys cinerea* (L.) Wight and Arn.	Munoa-mathoka	Shrub	0.02	Bark	Chewed and the extracts swallowed	Cough	Cough [64]
*Entada leptostachya* Harms	Mwaitha	Climber	0.10	Exudate, root	The exudate is applied into an injured eye. A root infusion is taken in case of food poisoning or a snake bite. A root poultice is used to message the body in case of an internal injury	Eye injury, food poisoning, snake bite, and internal injury	Snake bites, cuts, arrow poisoning and eye injuries [29, 34, 64]
*Indigofera lupatana* Baker f.	Muthika	Shrub	0.10	Bark	An infusion from the root bark is drunk for cough, diarrhea, and constipation. The root bark is alternatively chewed and the extracts swallowed	Cough, diarrhea, and constipation	Cough and stomach-ache [29, 34, 64]
*Senna occidentalis* (L.) Link	Musingili	Herb	0.04	Root	An infusion is drunk	Stomach-ache and diarrhea	Stomach-ache and diarrhea [1, 15]
*Tamarindus indica L*.	Kithumula	Tree	0.19	Leaf, fruit	An infusion from the fruit is drunk against tonsillitis. The tonsils are also massaged with leaf and fruit poultice. An infusion of fruits and leaves is drunk and bathed against smallpox, measles, and edema	Tonsillitis, smallpox, measles, and edema	Coughs, throat, measles, chicken pox, edema, sore throat, and oral thrush [1, 64]
*Tephrosia villosa* (L.) Pers.	Mwenyu	Herb	0.02	Root	A decoction is drunk	Malaria	Fever [44]
Loganiaceae							
*Strychnos henningsii* Gilg	Muteta	Tree	0.46	Bark, leaf	An infusion is drunk or powder from dried leaves and bark is infused in hot drinks	Malaria, constipation, pneumonia, kidney pains	Body pains, malaria, pneumonia, chest pains, and stomach-ache [1, 32, 34, 64]
Malvaceae							
*Grewia tembensis* Fresen.	Mutuva	Shrub	0.04	Bark	A decoction is drunk or the roots dried, ground into a powder, and infused in hot drinks	Reduced appetite, swollen diaphragm	Heartburn and cough [64, 65]
*Grewia tephrodermis* K.Schum.	Mulawa	Shrub	0.06	Roots, aerial parts	A root decoction is drunk against diarrhea, stomach-ache, and as an aphrodisiac in men. An infusion from the aerial parts is steam bathed for edema and skin rashes	Diarrhea, stomach-ache, aphrodisiac in men, edema, skin rashes	Dermatitis, diarrhea and restoring female fertility [1, 29, 64]
*Grewia villosa* Willd.	Muvu	Shrub	0.06	Root	An infusion is drunk	Diarrhea	Diarrhea, stomach-ache, and amoeboid dysentery [1, 64, 66]
*Sterculia africana* (Lour.) Fiori	Kiusya		0.13	Bark, aerial parts	An infusion is drunk	Diarrhea	Diarrhea and dysentery [1]
Meliaceae							
*Melia volkensii* Gürke	Mukau	Tree	0.04	Leaf	A decoction is drunk and bathed	Malaria, edema	Edema and malaria [34, 64]
Menispermaceae							
*Chasmanthera dependens* Hochst.	Usyiii	Liana	0.04	Stem	An infusion is drunk	Diarrhea	Reference [60]
*Cissampelos pareira L*.	Kutu kumwe	Climber	0.15	Root	An infusion is drunk	Diarrhea, stomach-ache	Stomach ailments [1, 44]
Moraceae							
*Ficus sycomorus L*.	Mukuyu	Tree	0.02	Exudate	Applied on tooth	Toothache	Toothache [1, 44]
Moringaceae							
*Moringa borziana* Mattei	Mululo	Shrub	0.17	Root	An infusion is bathed against edema and drunk for malaria and gonorrhea. The roots are alternatively dried and ground into a powder which is infused in hot drinks	Edema, malaria, and gonorrhea	References [71, 72]
Phyllanthaceae							
*Bridelia taitensis* Vatke and Pax ex Pax	Kyaanthya	Tree	0.02	Leaf	Is used as a bandage after application of herbal medicine in the form of a powder	Septic wounds	Reference [1]
Plumbaginaceae							
*Plumbago zeylanica L*.	Wala	Shrub	0.10	Root	A decoction is drunk, sometimes mixed with roots of *Moringa borziana*	Gonorrhea	Gonorrhea [64]
Rubiaceae							
*Hymenodictyon parvifolium* Oliv.	Mulinditi	Shrub	0.02	Root	An infusion is steam bathed	Edema	Edema [64]
*Tennantia sennii* (Chiov.) Verdc. and Bridson	Kisilingu	Shrub	0.08	Root	A decoction is drunk	Diarrhea, malaria, impotence, and infertility	Literature not found
Rutaceae							
*Vepris simplicifolia* (Engl.) Mziray	Mutuyu	Tree	0.10	Leaf, bark, root	An infusion is drunk and steam bathed	Edema, malaria	General body pains, malaria, and pleurisy [1, 17, 34, 36, 64, 66]
*Zanthoxylum chalybeum* Engl.	Mukenea	Tree	0.27	Bark, fruit	The root bark poultice is applied on an aching tooth as a remedy for toothache. An infusion from the bark or fruit is drunk against malaria, edema and cough. Alternatively, the bark, and the fruit are ground into a powder which is infused in hot drinks	Toothache, malaria, edema, and cough	Malaria, edema, cough, and toothache [1, 17, 29, 34, 64, 66]
Solanaceae							
*Solanum campylacanthum* Hochst.	Kitongu	Shrub	0.15	Root, fruit	An infusion from the root is drunk against diarrhea. Juice from a fruit is retained in the mouth as a remedy for toothache. Juice from a ripe fruit is applied topically on body parts infected with ringworms	Diarrhea, toothache, and ringworms	Stomach-ache, diarrhea, amoeboid dysentery, toothache, and ringworms [1, 32–34, 41, 64, 66, 73]
*Solanum tettense* Klotzsch	Mutongatongu	Shrub	0.02	Root	A decoction is drunk	Diarrhea and stomach-ache	Stomach-ache [64]
Vitaceae							
*Cissus aphyllantha* Gilg	Muvelengwa	Liana	0.06	Root	An infusion is used as a head wash in case of headache and drunk against diarrhea and pneumonia	Headache, diarrhea, and pneumonia	Diarrhea and amoeboid dysentery [34, 64]
*Cissus rotundifolia* Vahl	Itulu	Liana	0.02	Leaf	A poultice is used as a bandage	Septic wounds	Septic body swellings, boils, and used as ear drops [32, 44]
Xanthorrhoeaceae							
*Aloe secundiflora* Engl.		Herb	0.31	Exudate, inflorescence	An infusion of the exudate is drunk against malaria, cough, peptic ulcers, and a swelling of the diaphragm. The peduncle of the inflorescence is alternatively burnt to charcoal and ground into a powder which is licked or infused in hot drinks. An exudate from the leaf is dropped on septic wounds	Malaria, cough, peptic ulcers, swelling of the diaphragm, and wounds	Malaria, diarrhea, ulcers, swollen diaphragm, open wounds, and lack of appetite [1, 29, 34, 38, 64, 67, 73]
Zygophyllaceae							
*Balanites aegyptiaca* (L.) Delile	Kilului	Tree	0.08	Fruit	The pulp of a ripe fruit is eaten	Colds, cough, and kwashiorkor	Kwashiorkor, cough, and chest complaints [1, 29, 66]

**Table 4:** Classifications of diseases reported in Mutomo hill plant sanctuary and its environs.

Disease categories	Disorders reported	Number of plant taxa used
Infections or infestations	Gonorrhea, ringworms, tapeworms, pinworms, tuberculosis, yellow fever, malaria, measles, small pox, colds, pneumonia, and flu	33
Digestive system disorders	Diarrhea, peptic ulcers, stomach-ache, constipation, and toothache	24
Body abnormalities	Edema “body swelling”	19
Respiratory system disorders	Swollen diaphragm, cough, chest pains, tonsillitis, and swelling of the diaphragm	15
Body injuries	Boils, foreign objects pierced into the body, septic wounds, fresh cuts, and internal injuries	13
Genitourinary system disorders	Kidney pains, aphrodisiacs, impotence, and infertility	7
Muscular-skeletal system disorders	Back pains, pain in joints, and headache	4
Nutritional disorders	Kwashiorkor, reduced appetite	4
Sensory system disorders	Eye problems, earache	3
Skin/subcutaneous cellular tissue disorders	Warts, skin rashes	2
Circulatory system disorders	Hypertension	1
Poisonings	Snake bites, food poisoning	1
Ethnoveterinary applications	Diarrhea in livestock, tick infestation, eye problems, liver diseases, lung diseases	12

**Table 5:** Plant species with ethnoveterinary applications in Mutomo hill plant sanctuary and its environs. Where possible, literature citing ethnoveterinary uses of the reported plant species has been indicated.

Plant species	Part used	Ethnomedicinal applications	Disease treated	Reported ethnoveterinary uses
*Commiphora baluensis*	Bark	An infusion	Diarrhea in poultry	Diarrhea in chicken [64]
*Commiphora habessinica*	Exudate	Applied on areas infested with ticks	Deticking agent in goats and cattle	Is antiseptic [64]
*Boscia coriacea*	Leaves, root	An infusion is administered orally. The leaves are burnt and ground into a powder which is applied into the eyes	Diarrhea in goats and cattle. Partial blindness and eye injuries in cattle	Bile problems in poultry [40]
*Kleinia squarrosa*	Aerial parts	An infusion	Diarrhea in poultry	Literature not found
*Launaea cornuta*	Aerial parts	An infusion	Liver diseases in poultry	Diarrhea and coccidiosis in chicken [1, 64]
*Croton megalocarpus*	Leaves	An infusion	Liver disease in poultry	Diarrhea, dysentery, and swollen heads in poultry [74]
*Albizia anthelmintica*	Bark	An infusion is administered orally. Dried bark is ground into a powder which is applied into eyes	Liver diseases in poultry, eye injuries in cattle	Deworming and diarrhea in livestock [64]
*Plumbago zeylanica*	Roots, aerial parts	The roots are burnt and ground into a powder which is applied into injured eyes. An infusion from aerial parts is administered orally in poultry	Eye injury in cattle, lung and liver diseases in poultry	Literature not found
*Cissus quadrangularis*	Stem	An infusion is administered orally. The plant is sometimes mixed with the bark of *Commiphora baluensis* and *Albizia anthelmintica*	Diarrhea in poultry	Gall diseases, east coast fever, lung trouble, and diarrhea in cattle [64, 65]
*Cissus rotundifolia*	Leaves	An infusion	Diarrhea in poultry	Bloat, black quarter and anaplasmosis [1]
*Aloe secundiflora*	Exudate	An infusion	Diarrhea in poultry	Coccidiosis in chicken and diarrhea in livestock [1, 40, 64]

## Data Availability

The data used to support the findings of this study are included within the article.
